# Treatment of male and female spontaneously hypertensive rats with TNF-α inhibitor etanercept increases markers of renal injury independent of an effect on blood pressure

**DOI:** 10.1186/s13293-022-00424-4

**Published:** 2022-04-12

**Authors:** Elizabeth C. Snyder, Mahmoud Abdelbary, Ahmed El-Marakby, Jennifer C. Sullivan

**Affiliations:** 1 Department of Physiology, Medical College of Georgia at Augusta University, 1459 Laney Walker Blvd CB-2204, Augusta, GA 30912 USA; 2 Department of Oral Biology, Medical College of Georgia at Augusta University, Augusta, GA USA

**Keywords:** Sex, Gender, Inflammation, Kidney

## Abstract

Hypertension remains the leading risk factor for cardiovascular disease. Young females tend to be protected from hypertension compared with age-matched males. Although it has become increasingly clear that the immune system plays a key role in the development of hypertension in both sexes, few studies have examined how cytokines mediate hypertension in males versus females. We previously published that there are sex differences in the levels of the cytokine tumor necrosis factor (TNF)-α in spontaneously hypertensive rats (SHR). The goal of this study was to test the hypothesis that TNF-α inhibition with etanercept will lower BP in male and female SHR. However, as male SHR have a more pro-inflammatory status than female SHR, we further hypothesize that males will have a greater decrease in BP with TNF-α inhibition than females. Young adult male and female SHR were administered increasing doses of the TNF-α inhibitor etanercept or vehicle twice weekly for 31 days and BP was continuously measured via telemetry. Following treatment, kidneys and urine were collected and analyzed for markers of inflammation and injury. Despite significantly decreasing renal TNF-α levels, renal phospho-NFκB and urinary MCP-1 excretion, etanercept did not alter BP in either male or female SHR. Interestingly, treatment with etanercept increased urinary excretion of protein, creatinine and KIM-1 in both sexes. These results indicate that TNF-α does not contribute to sex differences in BP in SHR but may be vital in the maintenance of renal health.

## Highlights

- Etanercept treatment successfully decreased renal TNF-α levels in male and female SHR.
- Inhibition of TNF-α did not alter BP in SHR of either sex.
- Inhibition of TNF-α significantly elevated creatinine and KIM-1 levels in both male and female SHR, indicating TNF-α may be necessary for maintaining renal health.

## Introduction

Although hypertension is a prominent risk factor for developing cardiovascular disease (CVD), the mechanisms underlying the development of hypertension remain poorly understood. Hypertension is a common medical condition characterized by an increase in blood pressure (BP). An increasing number of studies have found pro-inflammatory cytokines produced by immune cells to be important contributors to the development of essential hypertension [1, 2], but few studies have examined the relative contribution of specific cytokines to BP control in males versus females.

Young females have a lower prevalence of hypertension compared to age-matched males both clinically and in numerous experimental models of hypertension, include spontaneously hypertensive rats [3] (SHR). Recent studies further indicate that while both sexes develop hypertension, the physiological and molecular mechanisms through which it develops often differ between males and females [4]. Importantly, there is increasing evidence that differing immunological profiles may contribute to sex differences in BP control. Sex differences in BP responses to angiotensin II and deoxycorticosterone acetate (DOCA)-salt administration have been shown to be mediated by immune cells [5–7]. Moreover, it has been suggested that the lower BP in females is related to the finding that females have a more anti-inflammatory and anti-hypertensive T cell profile than males in multiple experimental models of hypertension [8–10]. Direct neutralization of numerous pro-inflammatory cytokines have been shown to decrease systolic BP in male angiotensin II hypertensive rodents and spontaneously hypertensive models [1, 2, 11, 12]. However, there is less evidence available on the contribution of pro-inflammatory cytokines to hypertension in females or the role of cytokines in sex differences in hypertension.

The pro-inflammatory cytokine TNF-α has been implicated in BP control in angiotensin II, Dahl salt-sensitive, DOCA-salt male rat models of hypertension [13–16], and male SHR [12]. TNF-α has also been implicated in the development of hypertension in rodent models of preeclampsia [17] and in a female mouse model of systemic lupus erythematosus [18], supporting the hypothesis that TNF-α also mediates hypertension in females. Our group has previously published that female SHR have greater TNF-α levels in urine and mesenteric arteries compared to males [19], while circulating levels of TNF-α are higher in male SHR [20]. Despite data indicating TNF-α is a potent moderator of the processes governing hypertension development [21–24], the role of TNF-α in BP control in female SHR has not previously been examined. The current study was designed to test the hypothesis that TNF-α inhibition with etanercept will lower BP in male and female SHR. However, as male SHR have a more pro-inflammatory status than female SHR [4, 8, 25], we further hypothesize that males will have a greater decrease in BP with TNF-α inhibition than females.

## Materials and methods

### Animals

Nine-week-old age-matched male and female SHR were received from Envigo Laboratories (Indianapolis, IN). Rats were housed in temperature- and humidity-controlled, light-cycled quarters and fed standard rat chow ad libitum. All experiments were conducted in accordance with the National Institutes of Health Guide for the Care and Use of Laboratory Animals and with the approval of the Augusta University Institutional Animal Care and Use Committee. At 10 weeks of age, subsets of rats were implanted with intra-arterial telemeters for the measurement of mean arterial pressure (MAP) and allowed 5 days to recover before a baseline measurement was taken. At 12 weeks of age, rats began treatment with either the TNF-α inhibitor etanercept or vehicle via intraperitoneal injection twice weekly for 31 days. Based on previous studies in the literature, rats were treated with increasing doses of etanercept as follows: 0.4 mg/kg from days 1 to 12 [26], 0.8 mg/kg from days 13 to 22 [17], and 1.6 mg/kg from days 23 to 31 [15, 27, 28]. Rats were placed in metabolic cages to allow for a 24-h urine collection prior to initiating treatment with etanercept and at the end of the study. Rats were then anesthetized with 2% isoflurane and euthanized by aortic exsanguination. Kidneys were harvested and snap-frozen in liquid nitrogen and stored at −80 °C for later analysis.

### Cytokine quantification

Cytokine levels were determined by ELISA in whole kidney homogenate. Levels of TACE (LSBio F22349), TGF-β (MyBioSource 260302), TNF-α (MyBioSource 2507393) and phospho-NFκB (RayBiotech, PEL-NFKBP65-S536) were quantified in renal homogenates of etanercept-treated or vehicle control SHR and assayed according to the manufacturer’s instructions. Urinary MCP-1 excretion levels were determined by ELISA (BD Biosciences 555130).

### Assessment of renal injury

Creatinine and kidney injury molecule 1 (KIM-1) excretion were measured by ELISA (creatinine: Cayman Chemical 500701; KIM-1: MyBioSource MBS355395) in terminal 24-h urine samples according to manufacturer instructions. Proteinuria was measured using a Bradford assay using bovine serum albumin as a standard. Albuminuria was measured by Nephrat II ELISA (Ethos Biosciences NR002).

### Statistical analysis

All data are expressed as means ± SE. For all comparisons, *P* < 0.05 was considered statistically significant. Renal cytokines and markers of renal injury were compared using two-way ANOVA. BP data were analyzed by two-way repeated-measures ANOVA. Analyses were performed using GraphPad Prism version 9.2 software (GraphPad Prism Software, La Jolla, CA).

## Results

### Etanercept had no effect on BP in male or female SHR

**Fig. 1:**
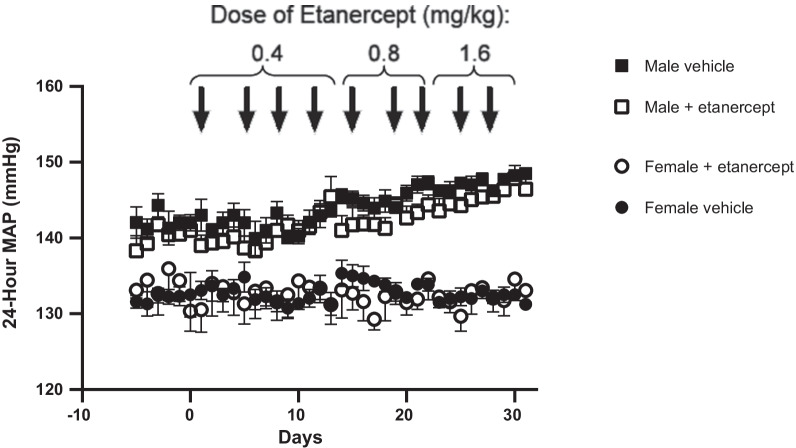
Male and female SHR were implanted with intra-arterial telemeters at 10 weeks of age, allowed to recover for 5 days, and then baseline measurements were taken for 1 week. Animals were then randomized to treatment with vehicle (*n* = 5/group) or increasing doses of etanercept (*n* = 5/group) and mean arterial pressure (MAP) was measured over a 31-day period. Comparisons were made using a two-way repeated-measures ANOVA

To determine the relative contribution of TNF-α on BP control in male and female SHR, rats were treated with increasing doses of the TNF-α inhibitor etanercept or vehicle and BP was continuously measured by telemetry (Fig. 1). Baseline BP data were as follows: BP in vehicle-treated males was 142 ± 2 mmHg, BP in etanercept-treated males was 141 ± 1 mmHg, BP in vehicle-treated females was 132 ± 1 mmHg, BP in etanercept-treated females was 134 ± 3 mmHg (two-way ANOVA: *P*_sex_ < 0.0001, *P*_treatment_ = 0.54, *P*_interaction_ = 0.086). BP at the end 0.4 mg/kg dose of etanercept was 145 ± 1 mmHg in vehicle-treated males, 141 ± 2 mmHg in etanercept-treated males, 133 ± 4 mmHg in vehicle-treated females, and 135 ± 2 mmHg in etanercept-treated females (two-way ANOVA: *P*_sex_ = 0.0014, *P*_treatment_ = 0.14, *P*_interaction_ = 0.64). BP at the end 0.8 mg/kg dose of etanercept was 147 ± 1 mmHg in vehicle-treated males, 144 ± 1 mmHg in etanercept-treated males, 132 ± 1 mmHg in vehicle-treated females, and 130 ± 2 mmHg BP in etanercept-treated females (two-way ANOVA: *P*_sex_ < 0.0001, *P*_treatment_ = 0.062, *P*_interaction_ = 0.88). BP at the end of the 1.6 mg/kg dose of etanercept was 149 ± 1 mmHg in vehicle-treated males, 146 ± 2 mmHg in etanercept-treated males, mmHg in vehicle-treated females, and 133 ± 2 mmHg in etanercept-treated females (two-way ANOVA: *P*_sex_ < 0.0001, *P*_treatment_ = 0.94, *P*_interaction_ = 0.16). Males had a higher BP than females throughout the study (*P* < 0.05). Treatment with etanercept did not significantly lower BP in either male or female SHR. Indeed, BP of male SHR progressively increased over the course of the study in both vehicle control and etanercept-treated rats. BP of female SHR remained constant throughout the study regardless of treatment.

### Treatment with etanercept decreased renal TNF-α

**Fig. 2:**
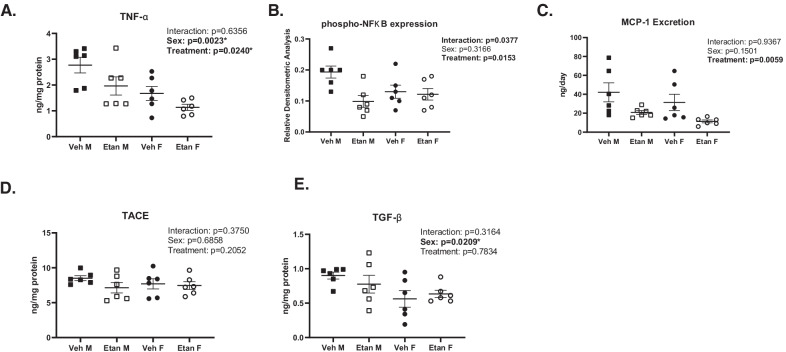
Quantification of TNF-α signaling and inflammatory cytokines in whole kidney homogenates of SHR. Rats were euthanized at 16 weeks of age following a 31-day treatment regimen of either vehicle (*n*=6/group) or increasing doses of etanercept (*n*=6/group). Kidneys were snap-frozen and homogenized for biochemical analysis of renal TNF-α (panel **A**), renal phosphorylation of NFκB (panel **B**), excretion of MCP-1 (panel **C**), renal TACE (panel **D**), and renal TGF-β (panel **E**). Data were compared using a two-way ANOVA. Values are mean ± SEM

To confirm the effectiveness and specificity of etanercept to inhibit TNF-α, TNF-α, NFκB, TACE and TGF-β were measured in renal homogenates and MCP-1 was measured in urine from vehicle and etanercept-treated male and female SHR (Fig. 2). Renal TNF-α levels were greater in males compared to females (*P*_sex_ = 0.0023). Etanercept lowered TNF-α levels in both sexes (*P*_treatment_ = 0.024), and the decrease was comparable in males and females (*P*_interaction_ = 0.64). TNF-α induces the phosphorylation of NFκB leading to translocation to the nucleus and increased transcription of inflammatory cytokines, including MCP-1. Etanercept decreased NFκB activation in male, but not female SHR (*P*_treatment_ = 0.0153; *P*_sex_  = 0.317; *P*_interaction_  = 0.038) and MCP-1 excretion in both sexes (*P*_treatment_  = 0.0059; *P*_sex_ = 0.15; *P*_interaction_  = 0.937). Renal TACE levels were comparable between all groups (*P*_sex_ = 0.6858, *P*_treatment_ = 0.2052, *P*_interaction_  = 0.3750). Males had higher renal TGF-β levels than females (*P*_sex_ = 0.0209), but TGF-β was not impacted by treatment with etanercept (*P*_treatment_  = 0.7834, *P*_interaction_ = 0.3164).

### Etanercept treatment increases indices of renal tubular injury

**Fig. 3:**
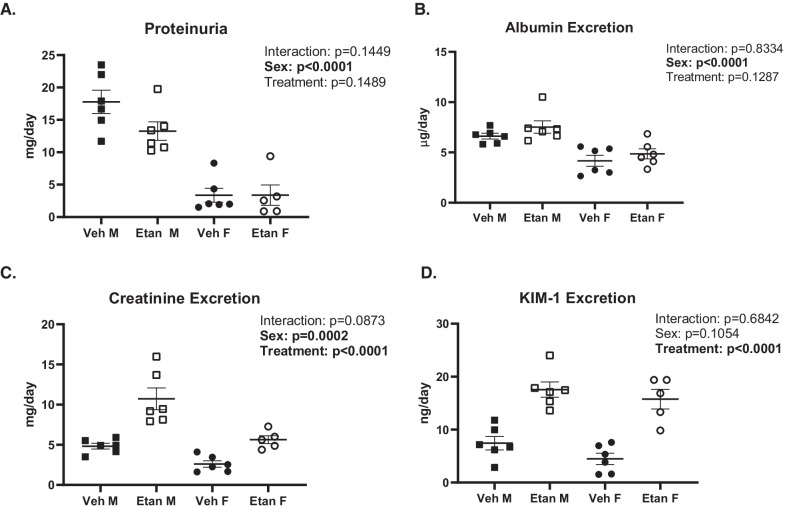
Quantification of renal injury in 16-week-old male and female SHR after a 31-day treatment regimen of either vehicle (*n* = 6/group) or increasing doses of etanercept (*n* = 5–6/group). Terminal urine samples were collected and assayed for common markers of renal damage, including proteinuria (panel **A**), albumin excretion (panel **B**), creatinine excretion (panel **C**), and KIM-1 excretion (panel **D**). Data were compared using a two-way. ANOVA. Values are mean ± SEM

Markers of renal injury were assessed by measuring urinary excretion of total protein, albumin, creatinine, and KIM-1, a marker of renal tubular injury (Fig. 3). Male SHR excreted more total protein (*P*_sex_ < 0.0001), albumin (*P*_sex_ < 0.0001), and creatinine (*P*_sex_ = 0.0002) than female SHR. KIM-1 excretion was comparable between the sexes (*P*_sex_ = 0.1054). Etanercept treatment did not affect total protein excretion (*P*_treatment_ = 0.1489; *P*_interaction_ = 0.1449) or albumin excretion in either male or female SHR (*P*_treatment_ = 0.1287; *P*_interaction_ = 0.8334). In contrast, etanercept increased creatinine (*P*_treatment_ < 0.0001) and KIM-1 excretion (*P*_treatment_ < 0.0001) in both sexes to a comparable degree (*P*_interaction_ = 0.0873 and *P*_interaction_ = 0.6842, respectively).

## Discussion

Despite an increasing number of studies implicating pro-inflammatory cytokines in the development of essential hypertension [1, 2], few studies have examined the relative contribution of specific cytokines to BP control in males versus females. The pro-inflammatory cytokine TNF-α has been implicated in BP control in both male [12–14, 16, 28] and female [17, 18] experimental models of hypertension. However, the main finding of the current study is that despite successfully decreasing renal TNF-α levels and downstream signaling cascades, etanercept did not alter BP in young, mature male or female SHR. Surprisingly, etanercept treatment was associated with increases in renal tubular injury as evidenced by increases in creatinine and KIM-1 in both male and female SHR. Our data suggest that renal tubular TNF-α is important in maintaining renal health and function in SHR.

While not intensively studied in animal models of hypertension, clinical studies have consistently found men to have higher circulating TNF-α levels compared to women [29, 30]. In line with this data, T cells from female mice demonstrate lower secretion of TNF-α than T cells from male mice in vitro [5], and we previously published that there are sex differences in TNF-α in SHR. Female SHR have greater TNF-α in the mesenteric arterial bed and urine versus males [19], while circulating levels of TNF-α are higher in male SHR [20]. As indicated in the current study, male SHR also have greater renal levels of TNF-α compared to female SHR. Based on known sex differences in BP in SHR, where males have a higher BP compared to females [4, 31], additional studies examined the contribution of TNF-α to BP control in SHR.

To assess the contribution of TNF-α on BP control in young, adult male and female SHR, rats were randomized to increasing doses of etanercept. Etanercept is a widely prescribed therapeutic which directly binds to TNF-α and prevents it from activating relevant cellular receptors [32]. The dose of etanercept was chosen based on previous studies in the literature, which have shown that daily treatment with 1.25 mg/kg etanercept decreases renal TNF-α levels and downstream inflammation in male DOCA-salt rats [15], attenuates angiotensin II-induced increases in MCP-1 [28], and attenuates hypertensive middle cerebral artery remodeling in male stroke-prone SHR [27]. Lower doses of etanercept (0.3 mg/kg/day) have also been shown to decrease relative heart wall thickness and increased cardiac reserve and BP in male SHR when measured via tail-cuff [26]. However, a noted limitation of the current study was the lack of measurement of circulating TNF-α levels.

Treatment with etanercept did not lower BP in young, adult SHR with established hypertension. There is some controversy in the literature regarding anti-hypertensive effects of etanercept. Etanercept, 1.25 mg/kg per day via subcutaneous osmotic minipump, delays the development of hypertension in male mice receiving angiotensin II plus a high-salt diet, although the effect is lost by day 12 of treatment [28]. Renal interstitial infusion etanercept (0.25 mg/kg/day) also attenuates high-salt diet induced increases in BP in male Dahl salt sensitive rats [14]. Etanercept (0.8 mg/kg weekly via ip or sc injection) also significantly lowered BP in 30-week-old female NZBWF1 mice, a model of systemic lupus erythematosus [18] and resulted in a small, but significant decrease in systolic but not diastolic BP pregnant female SHR stroke-prone rats, evident following 12 days of pregnancy [17]. However, 1.25 mg/kg per day etanercept via subcutaneous osmotic minipump did not alter the development of hypertension in male Sprague–Dawley rats in response to DOCA [15], and consistent with our findings, treatment of male SHR-stroke prone rats with etanercept (1.25 mg/kg ip daily) from 6 to 12 weeks of age did not alter systolic BP [27]. In addition, etanercept (0.3 mg/kg and 1 mg/kg; three times per week) did not alter BP in male 2-kidney and 1-Clip rats [33]. Taken together, these findings make it tempting to speculate that a BP phenotype in response to etanercept may be unveiled in SHR exposed to an additional stressor, such as salt.

In contrast to our findings in the current study, TNF-α blockade with infliximab (1.5 mg/kg/week, sc injection), a mouse chimeric neutralizing antibody, was found to decrease BP in male SHR compared to SHR receiving vehicle control [12]. However, treatment with infliximab did not significantly lower systolic BP until the 6th week of treatment. It is, therefore, possible that a longer regimen of etanercept treatment would decrease BP in male and female SHR. However, it has been demonstrated that infliximab does not bind directly to TNF-α in mice [34], suggesting that the anti-inflammatory and beneficial effects may be off-target. Therefore, etanercept may be preferred to study the contribution of TNF-α in preclinical models, since it has been shown to decrease TNF-α levels.

TNF-α induces inflammation, in part, via activation of NFκB signaling leading to increases in pro-inflammatory mediators, such as monocyte chemoattractant protein-1 (MCP-1). Therefore, the effectiveness and specificity of etanercept in the current study was confirmed by measuring decreases in TNF-α and MCP-1 with no effect on TACE or TGF-β. The impact of etanercept on immune cells and the source of TNF-α were not investigated in the current study. However, etanercept has been shown to decrease renal monocyte/macrophage infiltration in hypertension [18], and consistent with this we observed a decrease in MCP-1 excretion. T cells have also widely been linked to the development of hypertension and T cell production of TNF-α has been linked to the development of angiotensin II-dependent hypertension [13]. Etanercept has been shown to both increase pro-hypertensive Th17 cells [35, 36] and drive the expansion of anti-hypertensive T regulatory cells [37]. This differential impact on the T cell profile may account for the lack of a BP effect of etanercept in SHR.

Despite having no effect on BP, treatment with etanercept markedly increased urinary excretion of creatinine and KIM-1, suggesting a role for TNF-α in the control of renal tubular health and function in male and female SHR. Consistent with our finding, etanercept treatment has been associated with at least three clinical cases of acute kidney injury and subsequent renal damage [38–40]. TNF-α is involved in regenerative processes as well as inflammatory, thus its inhibition may be blunting regenerative mechanisms which typically protect against injury [16, 41]. Interestingly, renal TNF-α has been shown to be important in limiting increases in BP responses to salt and Ang II [42, 43], further supporting a key role for renal TNF-α in maintaining homeostasis. TNF-α suppresses intra-renal angiotensinogen expression via miR-133a, a salt-sensitive microRNA [43]. Additional studies have further shown that TNF-α receptor type 1 (TNFR1) mitigates intra-renal angiotensinogen production in response to Ang II plus high-salt [44]. Therefore, increases in renal tubular injury may be mediated by the loss TNF-α modulation of the intra-renal renin angiotensin system under normal physiological conditions in the SHR. Alternatively, this may be related to the effect of TNF-α on renal hypofiltration and diuresis [45]. Additional studies are needed to further understand the role of TNF-α and etanercept on renal health and function.

In summary, while we did not observe the hypothesized decrease in BP in SHR administered increasing dosages of etanercept, we found indications of renal damage in both male and female receiving etanercept treatment. Etanercept (i.e., Enbrel) is a common pharmaceutical approved for the treatment of rheumatoid arthritis. Therefore, even a slim probability for renal injury among a large patient population represents a risk of morbidity for a substantial number of patients. Further investigation is required to better understand how etanercept is increasing creatinine and KIM-1 excretion.

## Data Availability

All data collected for this study are included in this publication. If there are any additional questions, please contact the senior author.

## Abbreviations

CVD: Cardiovascular disease
BP: Blood pressure
SHR: Spontaneously hypertensive rat
TNF-α: Tumor necrosis factor alpha
MAP: Mean arterial pressure
TGF-β: Transforming growth factor beta
KIM-1: Kidney injury molecule 1
SE: Standard error
